# IL-8 and follicular fluid: insights into the mechanisms of endometriosis development

**DOI:** 10.17179/excli2025-8885

**Published:** 2026-01-02

**Authors:** A.T. Heinrich, A.L. Terres-Wurtz, E. Vacca, K. E. Tagscherer, B. Linek, S. Gebhard, A. Hasenburg, W. Brenner, R. Schwab

**Affiliations:** 1 Department of Obstetrics and Gynecology, University Medical Center of the Johannes Gutenberg University Mainz, Langenbeckstraße 1, 55131 Mainz, Germany; 2 Institute of Pathology, University Medical Center of the Johannes Gutenberg University Mainz, Langenbeckstraße 1, 55131 Mainz, Germany

**Keywords:** endometriosis, IL-8, follicular fluid, peritoneal microenvironment, CXCR1, CXCR2

## Abstract

Endometriosis is a common gynaecological condition characterised by the growth of endometrial-like tissue both within the muscular layer of the uterus and outside of it, affecting 10-15 % of women of reproductive age. This study investigated the role of the surrounding environment, specifically the potential role of follicular fluid (FF) and particularly its cytokine IL-8, in the growth and invasiveness of endometrial epithelial cells. Using the epithelial-like endometriotic cell line 12Z, we analysed cell viability and migration after exposure to three different FF pools at various dilutions. Our results demonstrated that FF increased cell viability, with the most significant effects at a 50 % (v/v) dilution after 24 h. Moreover, FF treatment reduced cell migration, while FF as a chemoattractant induced increased chemotactic cell migration, especially with pool FF1 as a chemoattractant. This FF pool contained the highest IL-8 concentration. Like FF, IL-8 showed a strong chemotactic effect, significantly reduced by inhibiting IL-8 receptors CXCR1 and CXCR2, confirming IL-8's role in chemotaxis. FF treatment induced the EMT marker N-cadherin and enhanced E-cadherin, indicating a hybrid cell EMT state. In conclusion, our study demonstrates that FF, particularly through IL-8 signalling, plays a crucial role in the pathogenesis of endometriosis by enhancing cell viability and influencing migration. These findings provide insights into how the local microenvironment contributes to disease progression.

See also the graphical abstract(Fig. 1).

## Abbreviations

AMH: Anti-Muellerian hormone

FF: Follicular fluid

FF1 to FF3: FF-sample pools 1 to 3

IL: Interleukin

PF: Peritoneal fluid

SR-M: Serum-reduced Medium (2 % FBS used here)

## Introduction

Endometriosis is a chronic gynaecological disorder where endometrial-like tissue grows outside the uterus. It affects 10-15 % of women of reproductive age (Giudice and Kao, 2004[12], Zondervan et al., 2020[41]). The exact causes are not fully understood, but various theoretical approaches have been proposed to explain the development of endometriosis lesions, including genetic/epigenetic dysregulation and immunological dysfunction (Koninckx et al., 2019[19], Kobayashi 2014[14]). In this context, the peritoneal cavity may influence superficial peritoneal lesions (Koninckx et al., 1998[17]). 

In women of reproductive age, the follicular fluid (FF) is a critical but physiological component of the peritoneal microenvironment (Somigliana et al., 2001[30]). The peritoneal fluid (PF) provides a dynamic microenvironment within the peritoneal cavity and includes plasma transudates, tubal fluid, retrograde menstrual residues, macrophage secretions, and ovarian exudates (Koninckx et al., 1980[18], Oral et al., 1996[27], Gazvani and Templeton, 2002[11]). Its volume temporarily peaks after ovulation, regulated by follicular activity and hormones (Syrop and Halme, 1987[33]). 

In detail, the increase in PF during the menstrual cycle is considered to be due to ovarian exudates, specifically FF (Koninckx et al., 1980[18], Bouckaert et al., 1986[4]). FF, a key component of PF, comprises secretions from the granulosa and theca cells and components from the blood vessels. It serves as a reservoir for essential nutrients and growth factors crucial for the oocyte's development (Sutton et al., 2003[32], Nagy et al. 2015[24], Basuino and Silveira, 2016[3]). Furthermore, it contains hormones, enzymes, anticoagulants, electrolytes, immune cells, and their products. In patients with underlying endometriosis and undergoing in vitro fertilization (IVF), analysis of the FF indicated an elevated presence of macrophages and increased concentrations of inflammatory cytokines such as IL-1ß, IL-6, IL-8, and IL-12 (Ryan et al., 1995[28], Lachapelle et al., 1996[20], Choi et al., 2015[5], Singh et al., 2016[29]).

The underlying mechanisms by which FF and its components influence endometriotic cells remain unclear. Elevated concentrations of the chemokine IL-8 have been detected in the FF of endometriosis patients, suggesting a potential role in the physiology of the endometrium and the pathogenesis of endometriosis (Singh et al., 2016[29]). IL-8 is well-known to exert a chemotactic effect as a ligand of the receptors CXCR1 and CXCR2, thus stimulating angiogenesis and directly influencing several physiological and pathological cellular processes (Koch et al., 1992[15], Zeilhofer and Schorr, 2000[38]). In this context, the IL-8 cytokine has regulatory functions in the adhesion and proliferation of ectopic endometrial stromal cells and the potential to suppress apoptosis (Arici, 2002[1], Li et al., 2012[22]). 

The general objective of this study was to investigate the role of FF, specifically its cytokine IL-8, in the growth and invasiveness of endometriotic cells. This research is novel in examining the functional behaviour of epithelial-like endometriotic cells exposed to FF, focusing on the chemotactic effects of IL-8 within the microenvironment of endometriosis. The central research question was: How does IL-8, found in elevated concentrations in FF, influence the viability and migration of endometriotic cells? Additionally, we aimed to understand the potential interplay between FF, IL-8, and the expression of cell adhesion molecules such as N-cadherin and E-cadherin. To address this, we employed in vitro cell culture models using the epithelial-like endometriotic cell line 12Z treated with different FF pools, followed by assays for cell viability, migration, and receptor expression. The findings of this study will contribute to a better understanding of the molecular mechanisms underlying endometriosis and could inform future therapeutic strategies to manage the disease.

## Materials and Methods

### Follicular Fluid collection and analysis

**Figure 2:**
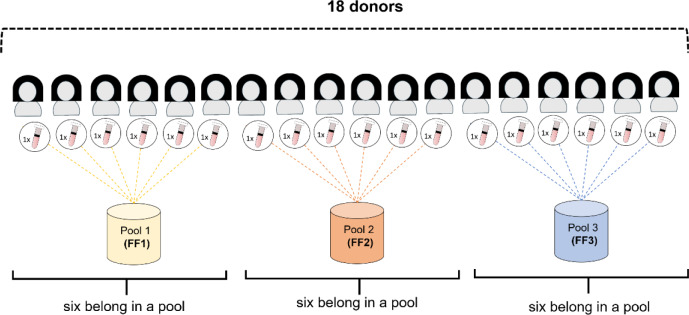
Preparation of follicle fluid (FF) pools. FF from 18 patients was obtained. To get a sufficient amount of FF for all experiments, equal amounts of FF from 6 donors were randomly pooled. FF samples from 18 donors thus resulted in three FF pools, each consisting of the FF from 6 donors.

FF was obtained from reproductive-age fertile women with a physiological ovarian reserve (AMH 1-8 ng/ml) who underwent IVF treatment (antagonist stimulation protocol) for male infertility. FF collection was approved by the local ethics committee (Landesärztekammer RLP, DE: 2020.15343) and conducted according to the Helsinki Declaration. After sample collection, the samples were anonymised. The FF was separated from cellular components by centrifugation at 800 x g for 10 min, followed by filtration. The amount of FF per donor was not sufficient for the experiments. To obtain a sufficient amount of FF for all experiments, equal amounts of FF from 6 donors were pooled at random. The FF samples from 18 donors, therefore, resulted in three FF pools, each consisting of the FF from 6 donors (Figure 2(Fig. 2)). These pools were designated as FF1 (pool 1), FF2 (pool 2), and FF3 (pool 3). Components of the FF were analysed by the Institute of Chemistry and Laboratory Medicine at the University Medical Center Mainz.

### Cell culture and cell treatment

12Z cells (Zeitvogel et al., 2001[39]) were obtained from Prof. Dr. Anna Starzinski-Powitz at Goethe University Frankfurt/Main, Germany. They were cultured in DMEM/F-12 Ham medium (Thermo Fisher Scientific, Waltham, USA), supplemented with 100 U/100 µg/mL of penicillin/streptomycin (Thermo Fisher Scientific, Waltham, USA) and 10 % Gibco™ Fetal Bovine Serum (FBS) (Thermo Fisher Scientific, Waltham, USA) at 37 °C in a 5 % CO_2_ atmosphere (HERAcell VIOS 160i CO2, Thermo Fisher Scientific, Waltham, USA). The culture medium was changed routinely every 2 to 3 days. 

A serum concentration of 2 % Gibco™ FBS (designated as SR-M, serum reduced medium) was used to assess cell viability and proliferation and limit FBS-derived effects. Pooled FF was added to the SR-M medium at the following concentrations: 10 % (v/v), 25 % (v/v), 50 % (v/v), and 75 % (v/v).

### MTT assay

The cellular viability was assessed using an MTT assay. Cells were plated at 3.5 x 10^3^ cells per well of a 96-well plate in quadruplicates for each data point and were cultured for 24 h. The culture medium was replaced with SR-M, and the cells were further incubated for 24 h. On the third day of culture, the cells were treated with the pooled FF solution, as described above and incubated for 24 h or 48 h. Afterwards, 10 µL MTT (5 mg/mL 3-(4,5 Dimethylthiazol-2-yl)-2,5-Diphenyl-Tetrazoliumbromide in DPBS) was added to each well. The plate was incubated at 37 °C in a 5 % CO_2_ atmosphere for 4 h. 100 µL solubilizing solution (10 % SDS in 0,01 M HCl) was added to dissolve the formazan crystals. After 4 h, a GloMax^®^ microplate reader (Promega, Walldorf, Germany) determined cell viability through the absorbance at 570 nm (reference wavelength: 750 nm).

### Chemotactic migration 

A Boyden chamber (Neuro Probe, Gaithersburg, USA) was utilized to assess cellular migration. The assay used polycarbonate membranes with a pore diameter of 8 µm and a surface area of approximately 7.8 mm^2^ per well. As chemoattractants, we used 10 µg/mL fibronectin (Thermo Fisher Scientific, Waltham, USA), prepared in 50 % v/v DPBS/SR-M, human recombinant IL-8 (CXCL8; R&D. Systems, Minnesota, USA) at concentrations of 200 pg/mL, 1 ng/mL, 100 ng/mL (diluted in 50 % v/v SR-M/DPBS) or FF diluted 1:2 in the SR-M. Cell suspension (in SR-M or FF/SR-M) was prepared at 3 x 10^5^ cells per mL concentrations. A total volume of 50 µL (1.5 x 10^4^ cells) of the cell suspension was added to each well on the upper part of the Boyden chamber and incubated for 16 h at 37 °C and 5 % CO_2_. To investigate whether IL-8 caused the migration, we applied the IL-8 inhibitor reparixin (Tocris Bioscience, Bristol, UK) at a 0.1 µM/mL concentration before IL-8 treatment. Subsequently, the membrane was washed twice, and cells were fixed with ethanol for 1 min. Non-migrated cells were removed from the upper side of the porous membrane using a cell scraper and rinsing with Weise buffer (Merck, Darmstadt, Germany). The migrated cells were then dried and fixed in ethanol for 1 min, followed by staining using Hemacolor-Kit (Merck, Darmstadt, Germany). Subsequently, the membranes were placed on microscope slides and covered with immersion oil. Cell migration was measured using a DMIL LED microscope (Leica, Wetzlar, Germany) at 400x magnification. Data were reported as total numeric values obtained from randomly selected fields (total membrane surface: 2.5 mm²) from three experiments conducted in quadruplicate.

### Flow cytometry

The levels of CXCR1 and CXCR2 expressions were measured using flow cytometry. 12Z cells at a confluency of approximately 50 % were pre-incubated with serum-free medium for 24 h. The cells were treated with 50 % (v/v) FF/SR-M or 50 % (v/v) DPBS/SR-M as a control for 24 h. Afterwards, the cells were rinsed with DPBS (Thermo Fisher Scientific, Waltham, USA), detached using trypsin (Thermo Fisher Scientific, Waltham, USA), and centrifugated for 5 min at 300 x g and room temperature. The cell pellet was washed twice, and cells were fixed using 4 % ROTI^®^ Histofix (Carl Roth GmbH + Co. KG, Karlsruhe, Germany). 1 x 10^6^ cells were suspended in DPBS + 1 % BSA (Sigma-Aldrich, St. Louis, USA) and incubated with a 1:10-diluted CXCR1/IL-8RA-PE antibody solution (R+D systems, Minneapolis, USA) or CXC2R/IL-8RB-PE antibody solution (R+D systems, Minneapolis, USA) for 60 min. Following this incubation, the cells were washed with 1.0 % BSA/DPBS buffer, centrifuged, and transferred to polypropylene tubes. The samples were kept in the dark at -4 °C until measurement. To quantify CXCR1 and CXCR2, a flow cytometer (BD Calibur, Becton Dickinson, Heidelberg, Germany) and CellQuest Pro software were used. A total of 15.000 events were analyzed for each determination.

### Immunocytochemical staining 

N-cadherin and E-cadherin expression levels were determined using the Dako REAL EnVision Detection System Kit (Dako/AGILENT, Hamburg, Germany). A total of 3.5 x 10^3^ cells were added to each chamber of an 8-chamber slide. The slide was incubated at 37 °C and 5 % CO_2_ for 24 h. SR-M replaced the medium, and the cells were incubated for another 24 h. On the third day of culture, the cells were treated with 50 % (v/v) SR-M diluted with either FF or DPBS for 24 h. Afterwards, the cells were fixed using 4 % ROTI^®^ Histofix (Carl Roth, Karlsruhe, Germany) for 1 h, washed, and their endogenous peroxidase was blocked with 3 % hydrogen peroxide (Merck, Darmstadt, Germany). The primary N or E-cadherin antibody (Cell Signaling, Boston, USA) (1:50) were incubated overnight in a humidity chamber at 4 °C. The slides were washed thrice with DPBS, and the secondary HRP rabbit/mouse antibody (Dako/AGILENT, Hamburg, Germany) was incubated at room temperature for 30 min. After washing with DPBS, the slides were stained with 3,3' diaminobenzidine for 4 min, washed with tap water, and then counterstained with Mayer's hematoxylin (Merck, Darmstadt, Germany). Dehydration was carried out using an ascending series of alcohol. Finally, the slides were embedded in Entellan^TM^ (Merck KGaA, Darmstadt, Germany) and analyzed under a DMIL LED microscope (Leica, Wetzlar, Germany). The negative control was carried out without the primary antibody.

### Statistics

Cell viability, proliferation, and migration were analysed in three independent experiments with quadruplicates. Standard deviations were calculated and presented as error bars. Statistical significance was determined with a two-tailed t-test with paired samples. Statistical significance was assumed at a p-value of < 0.05.

## Results

### Content of FF

To investigate the impact of follicular fluid (FF) on the development and progression of endometriosis, we collected 18 FF samples, which were then divided into three sample pools, each containing six samples from different patients. In these pools, 10 selected parameters were analysed and compared to reference values of blood serum (Table 1(Tab. 1); Reference in Table 1: Terres-Wurtz, 2023[34]). 

All FF pools demonstrated that IL-8 was more than three times higher in concentration than blood serum. The concentration of IL-6 also increased, but it exhibited greater variability than IL-8. As anticipated following in vitro fertilisation (IVF) treatment, the sample pools displayed elevated concentrations of estradiol, progesterone, and human chorionic gonadotropin (hCG) compared to average blood serum values. The highest levels of estradiol and hCG were observed in sample FF2, while the progesterone concentration was highest in sample FF3 (82.57 ng/mL). All other parameters analysed were within the serum concentration range (Table 1(Tab. 1)).

### The effect of FF on cell viability

**Figure 3:**
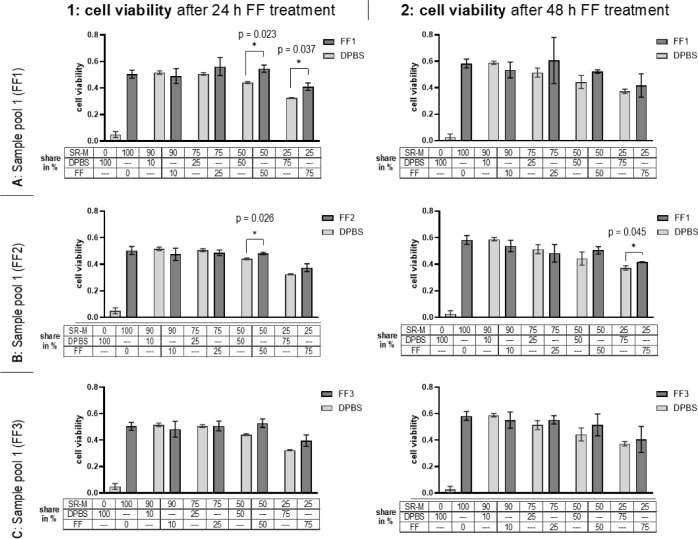
Treatment with follicle fluid (FF) significantly impacted cell viability. 12Z cells were treated with three pools of follicular fluid: FF1 (A), FF2 (B), and FF3 (C), at various concentrations (0, 10, 25, 50 and 75 % (v/v)), combined with serum-reduced medium (SR-M) for either 24 h (left) or 48 h (right). DPBS mixed with SR-M was used as a control. Cell viability was assessed using the MTT assay. Each bar represents the mean value and standard deviation from three independent technical replicates, each performed in quadruplicate. Significant results (p < 0.05) were determined using a two-tailed student's t-test and are indicated with an asterisk (*).

The impact of different concentrations of FF on the viability of 12Z cells over 24 h and 48 h was examined by MTT assay. Our results indicated a significant effect of FF on cell viability. Generally, cell viability decreased after 24 h with increasing FF concentration, with a notable reduction observed at 75 % (v/v) of FF compared to the DPBS controls (Figure 3(Fig. 3)).

Treatment with 10 % (v/v) of sample pool one (FF1) minimally reduced cell viability compared to DPBS, while FF in all other concentrations increased viability. Significant differences (p = 0.023; p = 0.037) were observed for the 50 % (v/v) FF and 75 % (v/v) FF treatments. Treatment with FF2 showed similar results as treatment with FF1, except for the 25 % (v/v) concentration, which resulted in a minimal reduction in viability compared to the control. Significant differences (p = 0.026) and increased cell viability were observed when the 12Z cells were treated with 50 % (v/v) FF. Treatment of the 12Z cells with 10 % (v/v) FF3 initially showed a slight reduction in viability compared to those treated with 10 % (v/v) DPBS. However, FF in all other concentrations increased viability compared to the DPBS control.

After 48 h, 12Z cells treated with 10 % (v/v) of all three FF pools had lower viability than the corresponding control. Cells treated with 25 % to 75 % (v/v) FF1 or FF3 were more viable than the DPBS controls. However, increasing FF concentration resulted in decreased cell viability. FF1 and FF3 induced the highest cell activity at 25 % (v/v) FF (0.61 and 0.55 relative units). The highest viability was achieved at a 50 % (v/v) FF concentration after treatment with FF2 (0.50 relative units). When 12Z cells were treated with 75 % (v/v) FF2, the viability values decreased compared to pure SR-M, but there was a significant 12 % increase in cell viability compared to the DPBS control (p = 0.044).

### Effect of FF on chemotactic cell migration

Cell migration plays a crucial role in the progression of malignancy, contributing to cell dissemination and metastasis. The motility and chemotactic potential of 12Z cells after FF treatment was investigated using a Boyden migration chamber. In both experiments, control (50 % v/v DPBS/SR-M) yielded an average cell migration of approximately 25 cells per mm^2^.

### Chemotactic cell migration after FF treatment

**Figure 4:**
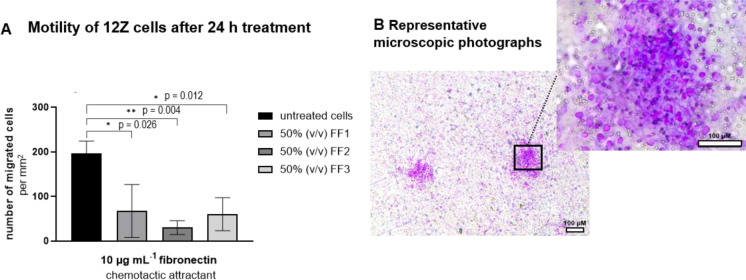
12Z cells' chemotactic migration was reduced after 24 h FF treatment. 12Z cells were treated with 50 % (v/v) follicular fluid (FF) diluted in serum-reduced medium (SR-M) for 24 h. Cell migration was assessed using a Boyden chamber over 16 h, with 10 µg/mL fibronectin as a chemotactic attractant. (A) Each bar represents the mean value and standard deviation from three independent technical replicates, each performed in quadruplicate. Significant results were analysed by a two-tailed student's t-test and marked as follows: p < 0.05 with (*), p < 0.01 with (**). (B) Microscopic photographs of migrated cells treated with FF2 are shown.

The motility of 12Z cells after 24 h treatment with 50 % (v/v) FF was analyzed using fibronectin as a chemotactic attractant. Compared with the untreated cells, a significant reduction in cell migration of the FF1, FF2, and FF3 treated cells was observed (p = 0.026; p = 0.004; p = 0.012, respectively). Untreated cells migrated to the chemotactic attractant at almost 200 cells per mm^2^, while 67 and 60 cells per mm^2^ were counted for the FF1 and FF3 treated cells. Cells treated with FF2 migrated with 30 cells per mm^2^, close to the negative control's average result. After passing through the porous membrane, the cells treated with FF come together in isolated clusters, while the untreated cells do not (Figure 4(Fig. 4)). This clumping of cells makes microscopic counting more difficult and could be a reason for the higher standard deviations compared to the negative control.

### Chemotactic cell migration with FF as chemoattractant

**Figure 5:**
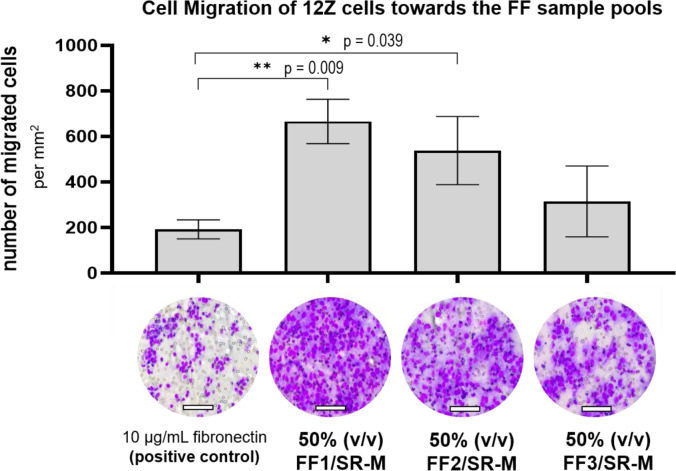
FF exhibited higher chemotactic potential on 12Z cells than fibronectin. Cell migration was assessed using a Boyden chamber. Three follicular fluid (FF) pools (FF1, FF2, FF3) were prepared by mixing them with DPBS in a 1:2 ratio (v/v) to serve as chemoattractants. 10 µg/mL fibronectin in 50 % (v/v) DPBS/SR-M was used as a positive control. Each bar represents the mean value and standard deviation from three independent technical replicates, each performed in quadruplicate. Demonstrated are the corresponding exemplary microscopic images. Significant results were analyzed by a two-tailed student's t-test and were marked as follows: p < 0.05 with (*), p < 0.01 with (**). Presented scale bars indicate 100 µM.

The chemotactic effect of FF on untreated 12Z cells, cultured in a 50 % (v/v) mixture of DPBS/SR-M, was evaluated in a Boyden chamber with 50 % (v/v) FF1-3/SR-M as chemoattractant. Fibronectin (10 µg/mL in 50 % v/v DPBS/SR-M) was utilized as a positive control. The results indicated that FF had a higher chemotactic potential on 12Z cells than Fibronectin (Figure 5(Fig. 5)). The most substantial potential was observed using FF1, inducing nearly a 3.5-fold increase in migration compared to Fibronectin (p = 0.009). This was followed by FF2 (2.8-fold, p = 0.039) and FF3 (1.6-fold, p = 0.0218).

### Effect of FF on the expression of E- and N-cadherin

Since we observed a reduced migration after FF treatment but a strong migration using FF as a chemoattractant, including noticeable cell aggregation, we investigated the EMT marker N- and E-cadherin. Epithelial cell migration occurs when cell-to-cell contacts are disrupted. As a result, epithelial cells lose their cell polarity and acquire invasive migratory properties similar to mesenchymal cells. The intracellular adhesion molecule E-cadherin is generally downregulated during this process, while the mesenchymal adhesion molecule N-cadherin is upregulated (Gaetje et al., 1995[8], 1997[9], Starzinski-Powitz et al., 1998[31]). 

**Figure 6:**
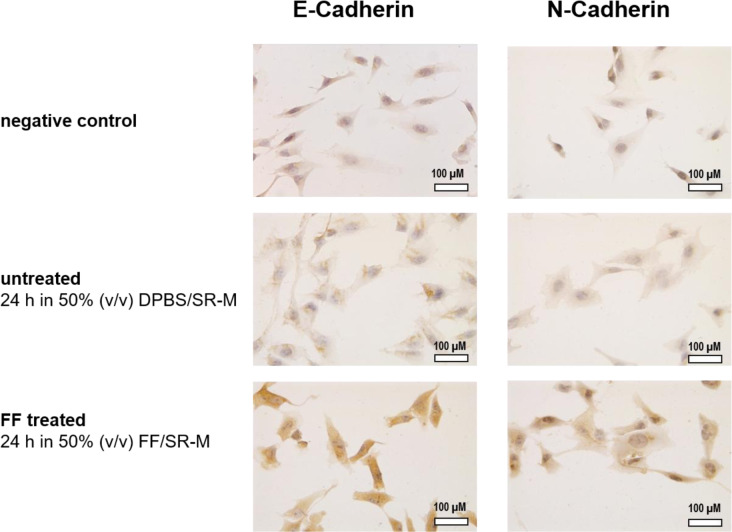
E-cadherin expression intensified after 24 h of treatment with FF, along with increased N-cadherin levels. 12Z cells grown on glass microscope slides were treated with 50 % (v/v) follicular fluid / serum reduced medium (FF/SR-M) or as control DPBS / serum reduced medium (DPBS/SR-M) for 24 h and immunocytochemically stained for E-cadherin and N-cadherin. The figure presents exemplary images of the technical negative control, untreated cells, and 12Z cells that received FF treatment.

The untreated cells showed weak expression of E-cadherin. After 24 h of treatment with FF, the expression of E-cadherin became more intense. N-cadherin, which was also slightly present in the untreated cells, exhibited a stronger expression after the cells were incubated with FF for 24 h (Figure 6(Fig. 6)).

### Impact of IL-8 in follicular fluid

FF1, as a chemotactic attractant, most strongly enhanced the migration ability of the 12Z cells, while FF3 induced comparatively lower migration. This finding was in line with the levels of IL-8 in the sample pools since IL-8 in FF1 was more concentrated than in FF3. A prerequisite for the impact of IL-8 on cell behaviour is the presence of IL-8 receptors CXCR1 and CXCR2. The receptor expression of both IL-8 receptors and the influence of FF was analysed by flow cytometry in untreated 12Z cells (50 % v/v DPBS/SR-M) and cells treated with 50 % (v/v) FF/SR-M.

**Figure 7:**
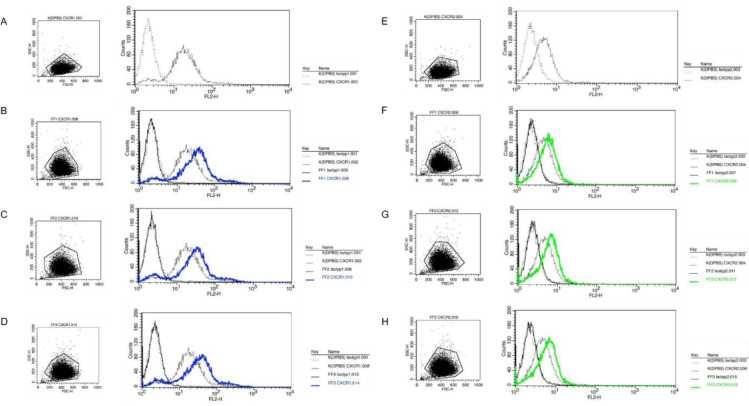
Treatment with 50 % (v/v) FF/SR-M increased CXCR1 levels more than CXCR2 levels in 12Z cells. 12Z cells were treated for 24 h with 50 % (v/v) follicular fluid / serum reduced medium (FF/SR-M) from three different follicular fluid pools (FF1, FF2, FF3) or with DPBS / serum reduced medium (DPBS/SR-M). The expression levels of (A) CXCR1 and (B) CXCR2 were assessed by flow cytometry. Phycoerythrin-conjugated antibodies specific to human CXCR1 and CXCR2, and a Phycoerythrin-conjugated IgG2A isotype control was employed for this analysis. A total of 15.000 cells were detected. The results are displayed as dot plots and histograms, showing the isotype controls (dashed line) alongside CXCR expression in untreated cells (black), CXCR1 (blue), and CXCR2 (green) in FF-treated cells.

The comparison of the shift in antibodies used to the respective isotype-specific antibodies showed that both untreated and FF-treated cells expressed CXCR1 and CXCR2 on their membranes. Treatment with 50 % (v/v) FF increased the expression of both receptors, with a more pronounced effect observed for CXCR1. Specifically, compared to untreated 12Z cells, FF treatment induced CXCR1 expression to 179 % for FF1, 173 % for FF2, and 186 % for FF3. In contrast, the expression of CXCR2 increased only by 20 % (FF1), 26 % (FF2), and 27 % (FF3), respectively (Figure 7(Fig. 7)).

### IL-8 as a chemoattractant in follicular fluid

The role of IL-8 in the chemotactic effect of FF on 12Z cells was tested using a Boyden chamber. Cells were treated with the CXCR1/2 inhibitor reparixin for 48 h, and IL-8 or FF were used as chemoattractants. Fibronectin (10 µg/mL in 50 % (v/v) DPBS/SR-M) was a positive control.

**Figure 8:**
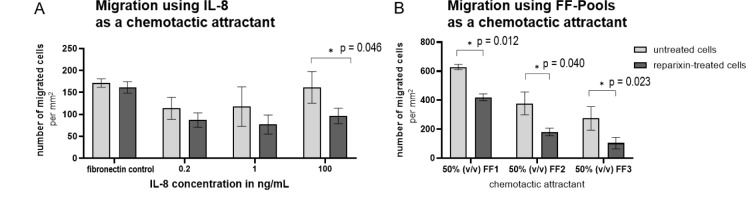
Pretreatment with the CXCR1/2 inhibitor reparixin reduced the chemotactic effect of IL-8 on 12Z cells and decreased cell migration towards FF. A Boyden chamber was utilised to assess the migration ability of 12Z. (A) IL-8 was used as a chemotactic attractant in concentrations of 0.2 ng/mL, 1 ng/mL, and 100 ng/mL in 50 % DPBS in serum reduced medium (DPBS/SR-M). 10 µg/mL fibronectin in 50 % (v/v) DPBS/SR-M was employed as a positive control. (B) 50 % (v/v) follicular fluid (FF) of three pools FF1, FF2, or FF3 in serum reduced medium (SR-M) served as a chemotactic attractant. Cells were pretreated with CXCR1 and CXCR2 inhibitor reparixin (0.1 µg/mL) to confirm the involvement of the IL-8 receptors. Each bar represents the mean value and standard deviation from three independent technical replicates, each performed in quadruplicate. Statistically significant results were analysed by a two-tailed student's t-test, comparing technical replicates of untreated versus reparixin-treated cells, and were marked as p < 0.05 with (*).

IL-8 showed a clear chemotactic impact on 12Z cells in a concentration-dependent manner. This effect was reduced when cells were pretreated with reparixin, an increasingly clear effect at higher IL-8 concentrations, with a 24 % reduction by using 200 pg/mL IL-8, 35 % by 1000 pg/mL IL-8, and 40 % by 100 ng/mL IL-8 (p = 0.046). Cell migration using fibronectin as a chemoattractant was not affected by reparixin (Figure 8(Fig. 8)).

To assess the significance of IL-8 in the FF, we used three FF pools (50 % (v/v) FF/SR-M) as chemoattractants and pretreated the 12Z cells with reparixin for 48 h. Our results indicated that cells showed strong movement in response to FF1 and weaker movement in response to FF3. Inhibiting CXCR1 and CXCR2 significantly reduced cell migration towards all three FF pools. The number of migrated cells decreased by 33 % for FF1 (p = 0.012), 52 % for FF2 (p = 0.040), and around 62 % for FF3 (p = 0.023) compared to the control (Figure 8(Fig. 8)).

See also the supplementary data.

## Discussion

The central research question of this study was to investigate the role of IL-8 in the chemotactic effects of FF on endometriotic cells, particularly in the context of FF's influence on cell migration and viability. Previous research (Singh et al., 2016[29], Iwabe et al., 1998[13]) suggested a potential link between IL-8 and endometriosis progression, but the exact mechanisms through which FF contributes to the invasiveness of endometrial cells remained unclear. Our findings demonstrated that FF, especially FF1 with higher IL-8 concentrations, significantly enhanced the chemotactic migration capability of the 12Z cells, supporting the hypothesis that IL-8 plays a critical role in this chemotactic process. These results not only confirm the involvement of IL-8 in FF-mediated migration but also expand on the current understanding of how FF influences endometriosis pathogenesis, bridging a critical gap in the literature regarding the molecular mechanisms behind endometrial cell behaviour in an inflammatory microenvironment.

This study demonstrated that FF significantly increased the viability of the 12Z cells, particularly FF1, which induced the most substantial chemotactic migration and contained the highest IL-8 concentrations. These findings support the hypothesis that IL-8 contributes to the chemotactic effects observed in FF, confirming its critical role in endometriosis pathogenesis. The results also indicate that IL-8 mediates its effects through the receptors CXCR1 and CXCR2, which were upregulated in response to FF treatment. These findings align with previous research suggesting that IL-8 is involved in cell proliferation and migration in endometrial stromal cells, and its role in driving disease progression via chemotaxis is now better understood (Iwabe et al., 1998[13], Opøien et al., 2013[26], Bahtiyar et al., 1998[2]).

Nevertheless, FF samples in this study were obtained from women undergoing IVF with hormonal stimulation. It is well-established that stimulated cycles, such as those induced by hCG, can alter the hormonal and cytokine profile of FF, potentially affecting the results. For example, compared to unstimulated cycles, hCG stimulation modifies progesterone, estradiol, and testosterone levels and affects the activity of follicular aromatase, which may influence the FF composition (Frederick et al., 1991[7], de los Santos et al., 2012[6]). Additionally, IVF stimulation protocols were associated with pronounced but statistically non-significant IL-8 concentration differences, where FF IL-8 levels were generally higher than those in serum (Kollmann et al., 2017[16]). This underscores the need for further studies using FF from natural cycles of endometriosis patients to confirm these findings under more physiologically relevant conditions, also by considering the physiologically stronger varying cytokine gradients in serum and FF. 

The variation in IL-8 levels across different FF pools (FF1, FF2, and FF3) also highlights the dynamic nature of FF. Specifically, FF1 and FF2 had higher IL-8 concentrations associated with more potent effects on cell viability and migration. Given the increase in peritoneal fluid volume from an average of 5 mL to 20 mL (Syrop et al., 1987[33], Koninckx et al., 1980[18], Bouckaert et al., 1986[4]), we decided to treat the cells with FF concentrations of 10 %, 25 %, 50 %, and 75 % (v/v) in DPBS. The physiological presence of other cytokines in the FF, such as IL-6, IL-12, and IL-1β, further complicates the interpretation, as these factors could interact with IL-8, enhancing its effects (Ryan et al., 1995[28], Lachapelle et al., 1996[20], Wunder et al., 2006[37], Choi et al., 2015[5]). Previous studies have shown that FF from women with endometriosis exhibits elevated IL-6 and IL-8 levels, which are likely to influence endometrial cell behaviour further and contribute to the disease progression (Ryan et al., 1995[28], Lachapelle et al., 1996[20], Choi et al., 2015[5], Singh et al., 2016[29]). Similar to our results, Bahtiyer et al., demonstrated effects on cell viability after an incubation period of 48 h with FF solely from endometriosis patients (Bahtiyar et al., 1998[2]).

FF in this study consistently promoted cell migration despite these variations, with the most potent chemotactic effects observed in FF1. This reinforces the notion that FF provides a favourable environment for cell survival and plays an active role in promoting the invasive characteristics of endometriotic cells. Furthermore, treating 12Z cells with FF induced the expression of N-cadherin, which is associated with EMT, a key process in the metastasis of cancerous cells and likely a crucial mechanism in endometriosis progression. These results suggest that FF facilitates the acquisition of more aggressive migratory phenotypes through EMT and highlights the potential for therapeutic interventions targeting these pathways to limit disease spread (Wilson et al., 1994[36], Zeitvogel et al., 2001[39], Olkowska-Truchanowicz et al., 2013[25]).

Bahtiyer's research showed that treating 12Z cells with 50 % FF (v/v) in DPBS enhances cell viability, creating a favourable environment for endometriotic cells (Bahtiyar et al., 1998[2]). However, in this study, 12Z cell viability decreased when treated with 75 % (v/v) FF in DPBS. The fluid's viscosity becomes a key factor at higher concentrations, such as 100 % (v/v) FF (data not shown). A possible explanation for this effect is that FF contains lipids, fatty acids, proteins, hormones, enzymes, growth factors, and metabolites. When the FF is too viscous, nutrient uptake by the cells is impaired, with negative consequences on cell growth and proliferation, subsequently leading to cell death. Additionally, the metabolic waste products presented in higher FF concentrations may further reduce cell viability. These factors explain why we avoided using 100 % (v/v) FF in our experiments and why 75 % (v/v) FF resulted in lower cell viability than lower concentrations.

Moreover, FF from individuals with inflammatory conditions, such as endometriosis, often contains elevated levels of cytokines, particularly IL-6 and IL-8. This may explain the difference in FF2, which contained samples from individuals with probably higher inflammatory conditions, as shown by the increased IL-6 levels. Since IL-8 levels were relatively consistent across the sample pools, we focused our research on this cytokine. Treatment with 50 % (v/v) FF1 in DPBS resulted in the most significant difference in cell viability after 24 and 48 h. FF1 and FF2 had the highest IL-8 concentrations, supporting that higher cytokine concentrations can significantly enhance cell viability. However, this was not the case for IL-6; despite high IL-6 concentrations in FF2, no effect on cell viability was observed.

The 12Z cells are classified as "epithelial-like," a cell type that is transitional between undifferentiated progenitor cells and fully differentiated cells (Zeitvogel et al., 2001[39]). Microenvironmental factors can influence this differentiation process, making it unclear how FF explicitly affects the migration of these cells. Our study showed a significant reduction in migration ability, suggesting stronger cell-cell interactions. Additionally, we noticed the formation of cell clusters during the migration test. We hypothesize that FF stimulates the expression of E-Cadherin, promoting a mesenchymal-epithelial transition (MET) that leads to the development of terminally differentiated epithelial cells. One possibility arising from this work is that these cell clusters make the cells less recognizable to the immune system. With enhanced and intact cell-cell communication, uncontrolled cell growth may occur. This could be particularly relevant in endometriosis, where patients exhibit altered immune responses to the accumulation of endometrial tissue. This includes impaired natural killer (NK) cell function, decreased phagocytosis, and increased regulatory T cells, all of which contribute to the reduced ability to clear cell debris (Wilson et al., 1994[36], Olkowska-Truchanowicz et al., 2013[25], Zhang et al., 2018[40]).

In addition to enhanced E-cadherin expression, treatment with FF resulted in increased N-cadherin expression, suggesting that the cells underwent an epithelial-mesenchymal transition (EMT). Components in FF may either promote this EMT process or, at the very least, stabilize transient hybrid state conditions in which cells display characteristics of both mesenchymal and epithelial forms. Moreover, FF may include factors that initiate the reverse process (MET) mentioned earlier. In the context of MET, E-cadherin is frequently moved to the cytoplasm. When cells are in these transient states, they demonstrate a blend of mesenchymal and epithelial characteristics.

The varying presence of cadherins also plays a role in cell migration. In this study, the observed decrease in migration after FF treatment may be linked to changes in integrin expression induced by FF treatment. Previous research showed reduced expression of integrin subunits in ectopic endometrial tissue compared to eutopic endometrium, which may affect the cells' migration ability (Gaetje et al., 2006[10]). Integrins bind to extracellular matrix components, facilitating coordinated cell migration through inside-out and outside-in signalling pathways. A previous study demonstrated that activating the FAK/AKT/MMP2 signalling cascade, initiated by the RGD sequence, promotes migration and invasion of stromal endometrial cells (Li et al., 2020[21]). Based on these findings, FF may influence the binding of the RGD-integrin complex, leading to reduced cell migration.

In our study, the endometriotic 12Z cells responded to environmental factors, including FF, which acted as a strong chemotactic attractant, leading to more significant cell migration than fibronectin. We observed the most substantial chemotactic potential in FF1 and the weakest in FF3. These results correlated with our cell viability findings, suggesting that varying IL-8 concentrations across the FF pools may influence cell viability and migration. Notably, we focused on IL-8 due to its consistent presence in the FF pools, with minimal fluctuations compared to IL-6.

Our flow cytometric analysis indicated that both IL-8 receptors, CXCR1 and CXCR2, may play a role in increased cell migration. After 24 h of FF treatment, both receptor expressions were upregulated, and CXCR1 showed a more substantial increase than CXCR2. Interestingly, untreated cells displayed CXCR1 expression about four times higher than CXCR2.

To assess the specificity of IL-8's chemotactic effect, we used reparixin, an IL-8 receptor inhibitor, during migration tests. While migration was more substantial with fibronectin as a chemoattractant, IL-8 also induced migration in a concentration-dependent manner. This effect was reversed upon application of reparixin, indicating that increased migration was primarily due to IL-8 binding to CXCR1 and CXCR2.

When we pretreated cells with reparixin, migration towards all three FF pools as chemoattractants significantly decreased. This confirms that IL-8 is a decisive chemotactic factor in FF, with FF3, the pool containing the lowest IL-8 concentration, resulting in the weakest migratory effect. However, despite the reduced migration, some cells continued to migrate, suggesting that other factors in FF, such as hormones, immune cells, growth factors, or transcription factors, also contribute to chemotactic migration.

IL-8 and its receptors, CXCR1 and CXCR2, are known to play a critical role in cancer progression (Luo et al., 2015[23]). In ovarian cancer cells (SKOV-3), IL-8 has been shown to initiate EMT by activating the WNT/β-catenin pathway (Wen et al., 2020[35]). Moreover, IL-8's connection to the activation of Focal Adhesion Kinase (FAK) signalling promotes stromal cell proliferation and invasion (Luo et al., 2015[23]). These findings, combined with our results, suggest that, similarly to carcinogenesis, IL-8 may contribute to the progression of endometriosis.

One of the strengths of this study is the use of the epithelial-like endometriotic cell line 12Z, one of the few endometriotic cell lines available, which allowed for a detailed examination of FF's effects in vitro, with careful control over FF concentrations. Furthermore, IL-8 receptor inhibitors provided robust evidence of IL-8's chemotactic role. However, this study's limitation is that the FF used was collected from IVF patients undergoing hormonal stimulation, which may not fully replicate the natural menstrual cycle conditions. For ethical reasons, it is not possible to obtain FF from healthy women who are not undergoing IVF. A corresponding comparison group could therefore not be included in the study. Another limiting factor is the lack of control experiments with IL-8. Unfortunately, the available amount of FF was no longer sufficient for these experiments. Further studies are planned to substantiate the significance of IL-8. Additionally, the effects of other cytokines and components within FF were not explored in detail, limiting the scope of our findings and offering space for further studies.

In summary, our study shows that IL-8 in follicular fluid increases the viability of endometriosis cells and also influences their migration, thereby potentially contributing to the progression of endometriosis. This shows that the local microenvironment can influence the progression of the disease and affect endometriotic processes. Further experiments are needed to consolidate our results and to examine the extent to which inhibition of IL-8 and its receptors could be considered as a therapeutic strategy.

## Declaration

### Notes

These results contribute to A.L.T.-W.'s doctoral thesis (Terres-Wurtz, 2023[34]). 

### Artificial Intelligence (AI) assisted technology

During the preparation of this work the authors used Grammarly-writing assistance in order to check grammar and increase the readability of the text. (After using this tool, the authors reviewed and edited the content as needed and take full responsibility for the content of the publication.)

### Conflict of interest

The authors state that they have no conflicts of interest that would affect their objectivity. This studied was not sponsored.

### Authors' contributions

A.T.H.: wrote the draft manuscript; A.L.T.-W. & E.V.: conceptualization, investigation, methodology, formal analysis, data curation; K.E.T.: formal analysis (flow cytometry) and technical support, B.L. & S.G.: technical support; A.H.: supervision & resources; W.B.: conceptualization, project administration, resources, supervision; R.S.: supervision, editing manuscripts draft and project administration. All authors have reviewed and approved the final version of the manuscript.

### Acknowledgments

We thank A. Starzinski-Powitz from Goethe-University, Frankfurt, Germany, for supplying the 12Z cells. 

## Supplementary Material

Supplementary data

## Figures and Tables

**Table 1:**
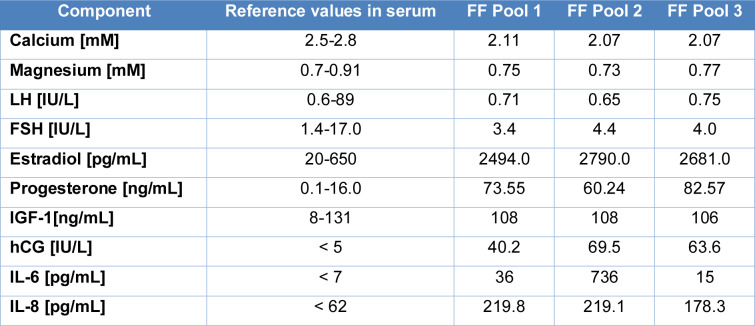
FF pools (FF1; FF2; FF3) clinical chemistry analysis. FF samples from women undergoing IVF for male infertility were pooled and analysed for selected cytokines, hormones, and minerals. LH: luteinising hormone; FSH: follicular-stimulating hormone; IGF-1: Insulin-like Growth Factor 1; hCG: Human chorionic gonadotrophin (data from Terres-Wurtz, 2023)

**Figure 1:**
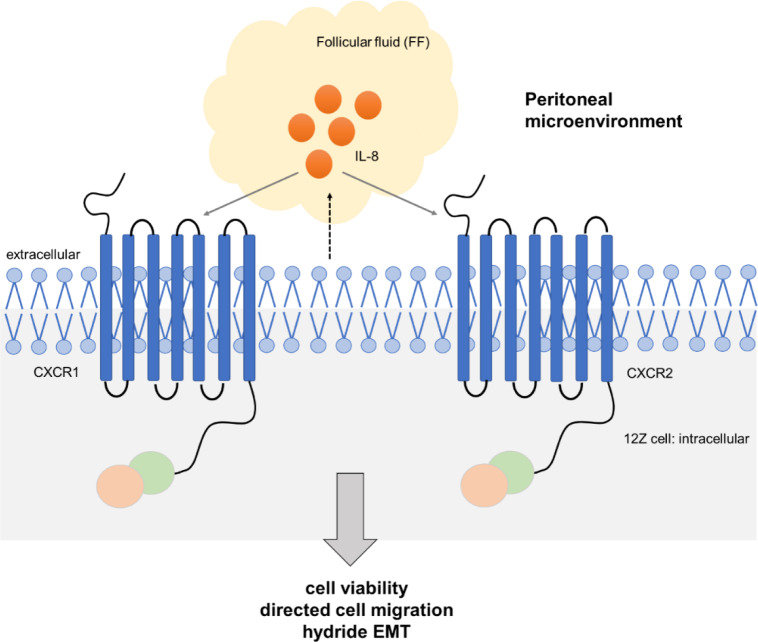
Graphical Abstract. IL-8: interleukin-8; EMT: epithelial-mesenchymal transition
