# Supplementary material for: IL-8 and follicular fluid: insights into the mechanisms of endometriosis development
Source: EXCLI J. 2026 Jan 2;25:1–17. doi: 10.17179/excli2025-8885 (PMC12901953; doi:10.17179/excli2025-8885)
Supplement: Supplementary data [file EXCLI-25-1-s-001.pdf]

EXCLI Journal 2026;25:1-17 – ISSN 1611-2156
Received: September 01, 2025, accepted: October 14, 2025, published: January 02, 2026

Supplementary data to:

Original article:

IL-8 AND FOLLICULAR FLUID: INSIGHTS INTO THE MECHANISMS OF ENDOMETRIOSIS DEVELOPMENT

A.T. Heinrich[1], A.L. Terres-Wurtz[1], E. Vacca[1], K. E. Tagscherer[2], B. Linek[1], S. Gebhard[1], A. Hasenburg[1], W. Brenner[1*], R. Schwab[1]

[1] Department of Obstetrics and Gynecology, University Medical Center of the Johannes Gutenberg University Mainz, Langenbeckstraße 1, 55131 Mainz, Germany
[2] Institute of Pathology, University Medical Center of the Johannes Gutenberg University Mainz, Langenbeckstraße 1, 55131 Mainz, Germany

* **Corresponding author:** Walburgis Brenner, Department of Obstetrics and Gynecology, University Medical Center of the Johannes Gutenberg University Mainz, Langenbeckstraße 1, 55131 Mainz, Germany, E-mail: brenner@uni-mainz.de

https://dx.doi.org/10.17179/excli2025-8885

This is an Open Access article distributed under the terms of the Creative Commons Attribution License (https://creativecommons.org/licenses/by/4.0/).

EXCLI Journal 2026;25:1-17 – ISSN 1611-2156
Received: September 01, 2025, accepted: October 14, 2025, published: January 02, 2026

Supplementary data to Figure 3: Cell viability is assessed using the MTT assay after exposure to the cells with various FF (Follicular Fluid) dilutions for 24 hours or 48 hours. The measured absorbance values from three independent experiments are shown. Values were used for statistical analysis (mean = average of the three independent experiments, SD = standard derivation, t-test = student's t-test) and were normalised to DPBS control.

24h viability								
		**RUN 1**	**RUN 2**	**RUN 3**	**Mean**	**SD**	**T.Test**	**%DPBS control**
**DPBS**	100%	0.0317	0.0745	0.0423	0.0495			
**SR-M**	100%	0.4876	0.4869	0.5393	0.5046			
**DPBS**	10%	0.5025	0.5190	0.5278	0.5165	0.0128		
	25%	0.5065	0.4955	0.5172	0.5064	0.0109		
	50%	0.4461	0.4335	0.4435	0.4410	0.0066		
	75%	0.3268	0.3266	0.3196	0.3244	0.0041		
**FF1**	10%	0.4513	0.4638	0.5550	0.4900	0.0566	0.4282	94.8803
	25%	0.5461	0.5031	0.6362	0.5618	0.0679	0.2363	110.9349
	50%	0.5679	0.5536	0.5155	0.5457	0.0271	**0.0234**	123.7323
	75%	0.3786	0.4305	0.4215	0.4102	0.0277	**0.0372**	126.4566
**FF2**	10%	0.4486	0.4491	0.5278	0.4752	0.0456	0.1901	92.0015
	25%	0.5005	0.4972	0.4624	0.4867	0.0211	0.3811	96.1053
	50%	0.4874	0.4858	0.4726	0.4819	0.0081	**0.0257**	109.2684
	75%	0.3498	0.4071	0.3666	0.3745	0.0295	0.0952	115.4484
**FF3**	10%	0.4623	0.4351	0.5504	0.4826	0.0603	0.3874	93.4407
	25%	0.5001	0.4733	0.5473	0.5069	0.0375	0.9776	100.0967
	50%	0.4930	0.5550	0.5374	0.5285	0.0320	0.0568	119.8273
	75%	0.3524	0.4381	0.4001	0.3969	0.0430	0.1020	122.3551

48h viability								
		**RUN 1**	**RUN 2**	**RUN 3**	**Mean**	**SD**	**T.Test**	**%DPBS control**
DPBS	100%	0.0117	0.0165	0.0536	0.0273	0.0229		
SR-M	100%	0.5486	0.5851	0.6151	0.5829	0.0333		
DPBS	10%	0.5742	0.5953	0.5962	0.5886	0.0125		
	25%	0.5482	0.5148	0.4792	0.5141	0.0345		
	50%	0.4996	0.4211	0.4082	0.4430	0.0495		
	75%	0.3541	0.3798	0.3851	0.3730	0.0166		
FF1	10%	0.5058	0.4935	0.6017	0.5337	0.0593	0.2255	90.6693
	25%	0.5745	0.5225	0.7215	0.6061	0.1743	0.3459	117.9111
	50%	0.5100	0.5360	0.5192	0.5217	0.0132	0.1478	117.7836
	75%	0.3154	0.4799	0.4543	0.4166	0.0885	0.4094	111.6735
FF2	10%	0.5187	0.5068	0.5870	0.5375	0.0433	0.1565	91.3169
	25%	0.5595	0.4449	0.4443	0.4829	0.0664	0.3163	93.9358
	50%	0.5168	0.4725	0.5252	0.5048	0.0283	0.1692	113.9606
	75%	0.4169	0.4136	0.4196	0.4167	0.0030	0.0445	111.7144
FF3	10%	0.5344	0.5009	0.6197	0.5517	0.0613	0.3918	93.7247
	25%	0.5742	0.5166	0.5691	0.5533	0.0318	0.2737	107.6306
	50%	0.4301	0.5208	0.5943	0.5151	0.0823	0.4382	116.2790
	75%	0.2913	0.4707	0.4529	0.4050	0.0989	0.5732	108.5638

EXCLI Journal 2026;25:1-17 – ISSN 1611-2156
Received: September 01, 2025, accepted: October 14, 2025, published: January 02, 2026

Supplementary data to Figure 4: Cell migration results, which were utilised with a Boyden-migration chamber and fibronectin as a chemoattractant (visualised diagrams were normalized by qmm). The table indicates the number of cells counted for each membrane.

data per 2.5 qmm

Sample	membrane 1	membrane 2	membrane 3
**negative control**	76.5000	103.5000	93.5000
**positive control**	559.5000	494.0000	425.2500
**50% (v/v) FF1**	339.0000	100.5000	66.5000
**50% (v/v) FF2**	119.7500	47.7500	59.0000
**50% (v/v) FF3**	253.5000	75.0000	121.7500

EXCLI Journal 2026;25:1-17 – ISSN 1611-2156
Received: September 01, 2025, accepted: October 14, 2025, published: January 02, 2026

Supplementary data to Figure 5: The number of migrated cells per area of the 12Z cells towards the three FF sample pools and to the positive control. The table indicates the number of cells counted for each membrane.

data per 2.5 qmm

Sample	membrane 1	membrane 2	membrane 3
**negative control**	58.0000	18.5000	34.0000
**positive control**	592.0000	384.5000	464.2500
**50% (v/v) FF1**	1916.7500	1650.2500	1430.0000
**50% (v/v) FF2**	1753.5000	1261.7500	1019.5000
**50% (v/v) FF3**	1233.5000	601.2500	526.7500

EXCLI Journal 2026;25:1-17 – ISSN 1611-2156
Received: September 01, 2025, accepted: October 14, 2025, published: January 02, 2026

Supplementary data to Figure 6: Immunhistochemical staining of E- and N-cadherin after 24 h FF treatment. Three independent technical replicas are shown. Figure A corresponds to figure 6 of the manuscript.

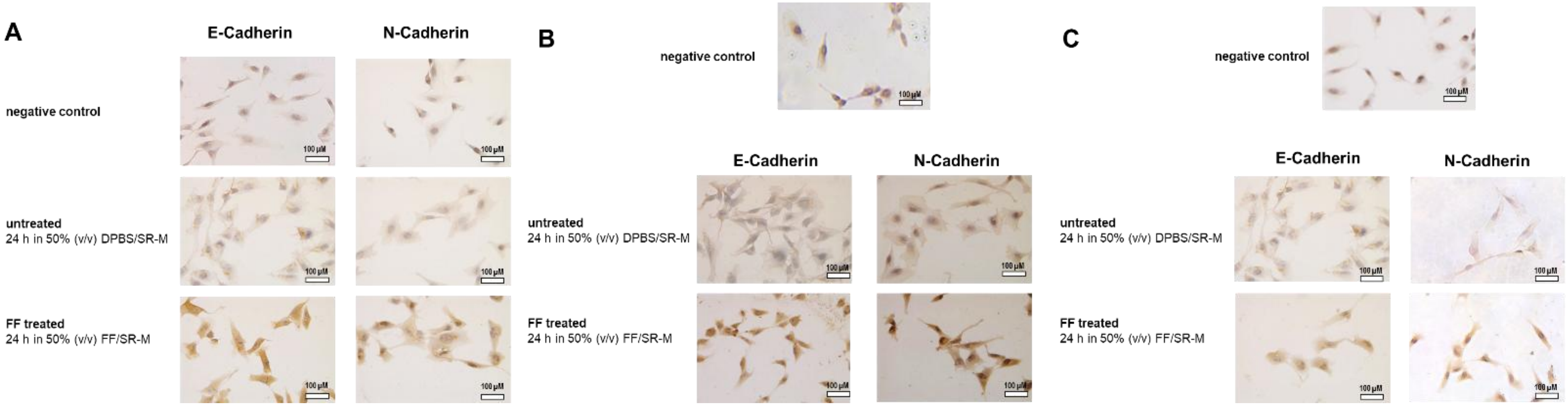

EXCLI Journal 2026;25:1-17 – ISSN 1611-2156
Received: September 01, 2025, accepted: October 14, 2025, published: January 02, 2026

Supplementary data to Figure 8: Raw data of the cell migration experiment utilised by the Boyden-chamber. In panel (A), IL-8 was used as a chemoattractant, while FF pools were used in panel (B). The table indicates the number of cells counted for each membrane.

data per 2.5 qmm

A

	membrane 1		membrane 2		membrane 3	
**Sample**	non treated	reparixin treated	non treated	reparixin treated	non treated	reparixin treated
**negative control**	63	20.25	18.25	18.25	14.25	47.5
**positive control**	423	396	454.75	376.5	407.5	440.25
**200pg/ml IL8**	312.5	262	328.25	183	212.75	208
**1000pg/ml IL 8**	420.5	252	259.75	146	203.5	178.25
**100ng/ml IL8**	448.75	287.25	463.24	236	300.75	199.75

data per 2.5 qmm

B

	membrane 1		membrane 2		membrane 3	
**Sample**	non treated	reparixin treated	non treated	reparixin treated	non treated	reparixin treated
**negative control**	35	63.25	53.25	38.25	46.75	43.3
**200pg/ml IL8**	1522.5	1107.5	1592.5	1055	1604.25	991.75
**1000pg/ml IL 8**	1168.25	475.75	870.75	506.75	800	381.75
**100ng/ml IL8**	910	354.25	633.25	275	516.75	155.75

EXCLI Journal 2026;25:1-17 – ISSN 1611-2156
Received: September 01, 2025, accepted: October 14, 2025, published: January 02, 2026

Normalisation & Statistics: Normalisation of the cell migration experiments: The number of migrated cells was calculated per 1 mm² and values were used for statistical analysis (mean = average of the three independent experiments, SD = standard deviation, and t-test = Student's t-test).

data per 2.5 qmm

Sample	membrane 1	membrane 2	membrane 3
negative control	76.5000	103.5000	93.5000
positive control	559.5000	494.0000	425.2500
50% (v/v) FF1	339.0000	100.5000	66.5000
50% (v/v) FF2	119.7500	47.7500	59.0000
50% (v/v) FF3	253.5000	75.0000	121.7500

normalized (per qmm)

cell migration after 24 h FF treatment

Sample	membrane 1	membrane 2	membrane 3	Mean	STD	T.Test
negative control	30.5714	41.3571	37.4286	36.4524	5.4587	
positive control	223.7857	197.6429	170.1429	197.1905	26.8243	
50% (v/v) FF1	135.5714	40.2143	26.5714	67.4524	59.3859	0.0255
50% (v/v) FF2	47.9286	19.0714	23.5714	30.1905	15.5255	0.0037
50% (v/v) FF3	101.3571	30.0000	48.7143	60.0238	36.9985	0.0121

data per 2.5 qmm

Sample	membrane 1	membrane 2	membrane 3
negative control	58.0000	18.5000	34.0000
positive control	592.0000	384.5000	464.2500
50% (v/v) FF1	1916.7500	1650.2500	1430.0000
50% (v/v) FF2	1753.5000	1261.7500	1019.5000
50% (v/v) FF3	1233.5000	601.2500	526.7500

normalized (per qmm)

cell migration towards FF

Sample	membrane 1	membrane 2	membrane 3	Mean	STD	T.Test
negative control	23.2000	7.4000	13.6000	14.7333	7.9607	
positive control	236.8000	153.8000	185.7000	192.1000	41.8685	
50% (v/v) FF1	766.7000	660.1000	572.0000	666.2667	97.4964	0.0087
50% (v/v) FF2	701.4000	504.7000	407.8000	537.9667	149.6003	0.0387
50% (v/v) FF3	493.4000	240.5000	210.7000	314.8667	155.3307	0.2182

data per 2.5 qmm

	membrane 1		membrane 2		membrane 3	
Sample	non treated	reparixin treated	non treated	reparixin treated	non treated	reparixin treated
negative control	63	20.25	18.25	18.25	14.25	47.5
positive control	423	396	454.75	376.5	407.5	440.25
200pg/ml IL8	312.5	262	328.25	183	212.75	208
1000pg/ml IL 8	420.5	252	259.75	146	203.5	178.25
100ng/ml IL8	448.75	287.25	463.24	236	300.75	199.75

normalized (per qmm)

cell migration in IL-8's way

	membrane 1		membrane 2		membrane 3		Mean		STD		T.Test
Sample	non treated	reparixin treated	non treated	reparixin treated	non treated	reparixin treated	non treated	reparixin treated	non treated	reparixin treated	
negative control	25.2	8.1	7.3	7.3	29.7	49.3	20.7333	21.5667	11.8492	24.0211	
positive control	169.2	158.4	181.9	150.6	163	176.1	171.3667	161.7000	9.6345	13.0664	
200pg/ml IL8	125	104.8	131.3	73.2	85.1	83.2	113.8000	87.0667	25.0537	16.1510	0.2476
1000pg/ml IL 8	168.2	100.8	103.9	58.4	81.4	71.3	117.8333	76.8333	45.0462	21.7348	0.1334
100ng/ml IL8	179.5	114.9	185.296	94.4	120.3	79.9	161.6987	96.4000	35.9692	17.5855	0.0464

data per 2.5 qmm

	membrane 1		membrane 2		membrane 3	
Sample	non treated	reparixin treated	non treated	reparixin treated	non treated	reparixin treated
negative control	35	63.25	53.25	38.25	46.75	43.3
50% (v/v) FF1	1522.5	1107.5	1592.5	1055	1604.25	991.75
50% (v/v) FF2	1168.25	475.75	870.75	506.75	800	381.75
50% (v/v) FF3	910	354.25	633.25	275	516.75	155.75

normalized (per qmm)

cell migration in IL-8's way (out of FF)

	membrane 1		membrane 2		membrane 3		Mean		STD		T.Test
Sample	non treated	reparixin treated	non treated	reparixin treated	non treated	reparixin treated	non treated	reparixin treated	non treated	reparixin treated	
negative control	14	25.3	21.3	15.3	18.7	17.32	18.0000	19.3067	3.7000	5.2877	
50% (v/v) FF1	609	443	637	422	641.7	396.7	629.2333	420.5667	17.6795	23.1833	0.0120
50% (v/v) FF2	467.3	190.3	348.3	202.7	320	152.7	378.5333	181.9000	78.1656	26.0369	0.0402
50% (v/v) FF3	364	141.7	253.3	110	206.7	62.3	274.6667	104.6667	80.7974	39.9678	0.0229
